# Activity-Based Prospective Memory in ADHD during Motor Sleep Inertia

**DOI:** 10.3390/s23115181

**Published:** 2023-05-30

**Authors:** Miranda Occhionero, Lorenzo Tonetti, Andreas Conca, Sara Giovagnoli, Giancarlo Giupponi, Marina Zoppello, Vincenzo Natale

**Affiliations:** 1 Department of Psychology “Renzo Canestrari”, University of Bologna, 40127 Bologna, Italy; miranda.occhionero@unibo.it (M.O.); lorenzo.tonetti2@unibo.it (L.T.); sara.giovagnoli@unibo.it (S.G.); 2 Division of Psychiatry, San Maurizio Hospital, 39100 Bolzano, Italy; andreas.conca@sabes.it (A.C.); giancarlo.giupponi@sabes.it (G.G.); 3 Child Neuropsychiatry Unit, Istituto di Ricovero e Cura a Carattere Scientifico Mondino Foundation, 27100 Pavia, Italy; marina.zoppello@gmail.com

**Keywords:** activity level, ADHD, adulthood, child development, prospective memory

## Abstract

Prospective memory (PM) is essential in everyday life because it concerns the ability to remember to perform an intended action in the future. Individuals diagnosed with attention deficit hyperactivity disorder (ADHD) often show poor performance in PM. Because age can be confounding, we decided to test PM in ADHD patients (children and adults) and healthy controls (children and adults). We examined 22 children (four females; mean age = 8.77 ± 1.77) and 35 adults (14 females; mean age = 37.29 ± 12.23) with ADHD, in addition to 92 children (57 females; mean age = 10.13 ± 0.42) and 95 adults (57 females; mean age = 27.93 ± 14.35) as healthy controls. Each participant originally wore an actigraph around the non-dominant wrist and was requested to push the event-marker at get-up time. To assess the efficiency of PM performance, we calculated the time elapsing between the end of sleep in the morning and the pushing of the event-marker button. The results showed lower PM performance in ADHD participants, regardless of age. However, the differences between ADHD and control groups were more evident in the children group. Our data seem to confirm that PM efficiency is compromised in individuals diagnosed with ADHD regardless of age, and agree with the idea of considering the PM deficit as a neuropsychological marker of ADHD.

## 1. Introduction

Attention-deficit hyperactivity disorder (ADHD) is a neurodevelopmental disorder characterized by symptoms of motor hyperactivity, impulsivity, and inattention. The prevalence of ADHD in childhood is high, i.e., around 5% [1], with a persistence in about half of the cases into adulthood [2,3,4].

It has been proposed that children with ADHD have difficulty maintaining an appropriate problem-solving set to attain future goals, reflecting an executive function deficit [5,6]. This executive deficit appears in several processes closely linked to prospective memory (PM) performance, resulting in failures to execute goal-directed activities in their everyday lives, as goals are often not achieved, and tasks left unfinished [7]. The PM function emerges early in childhood and becomes increasingly important in primary school [8]. However, a distinction must be made between different types of prospective memory. There are three types of PM [9]: (1) time-based PM, with an intention to perform the intended action at a specific time of day or after a specific amount of time; (2) event-based PM, when an external cue is necessary in order to activate the intention to perform the intended action; and (3) activity-based PM, with an intention to perform the intended action prior to or after an activity. Because time-based PM tasks are associated with top-down control mechanisms—in particular monitoring accuracy, subjective time estimation and self-initiated retrieval processes, which require executive resources with the involvement of prefrontal areas—they are usually considered more difficult compared with event-based PM tasks [10]. Because activity-based PM tasks occur at the end of a task and therefore do not require the interruption of an ongoing task, they are considered even easier [9]. Moreover, time-based prospective memory skills develop last, and the process has a greater need for attentional resources to strategically monitor the PM target time. This competence develops at the end of early childhood, when the executive functions and memory systems are well structured [11], alongside a maturation of the cognitive ability to make time estimations [12].

A debated issue in prospective memory studies is related to the ecological validity of the experimental data [13,14]. In the laboratory setting, it is very difficult to create tasks with the characteristics of complexity, importance, and timing like those that occur in everyday life. For example, in a typical time-based task, the time interval used to establish the correct execution is very narrow, minutes or even less. Similarly, an event-based task is considered correct only if performed in close coincidence with the cue event and in an activity-based task, the performance is correct only if performed at the end of a specific activity. This methodological approach produces an undoubted advantage in the control of the experimental variables. However, it poses some problems if we are interested in understanding the impact of PM in everyday life. Arriving at the station on time is a suitable behavior to avoid missing a train, but the time unit must be calibrated more widely, well beyond seconds. Taking a drug at 8.00 a.m. does not imply a prospective error if the execution is at 7.50 or 8.15, far more serious would be not taking the drug. In everyday behavior, it is certainly important to do things while meeting a certain deadline, be it self-generated (time-based) or connected to an external cue-event (event- or activity-based). However, the flexibility of behavior towards this deadline can be variable and does not necessarily compromise the success of prospective performance.

Another important aspect is linked to the development of prospective memory competencies. In children, PM assumes great importance in everyday life, for example, in planning school learning, social, or free time activities. Therefore, it is surprising that, to date, PM skills in children with typical development and with ADHD have been investigated in so few ecologically valid studies [15]. In this respect, Yang, Chan, and Shum [16] who examined event-, time, and activity-based PM simultaneously in typically developing children aged between 7 and 12, found that performance on the activity-based PM task was greater than on the event-based PM task, which was, in turn, better than performance on the time-based PM task [16]. Yang et al. [17] obtained the same results in a sample of 28 children with ADHD, showing the following percentages of accuracy: activity-based PM = 89%; event-based PM = 67%, time-based PM = 40% [17]. Furthermore, the performance of the ADHD sample was similar to the control group in all three PM tasks.

A recent study [18], which aimed to objectively clarify the role of sleep quality in activity-based PM, obtained similar results to Yang et al. [17]. In this study, a naturalistic activity-based PM task was used, i.e., remembering to press the actigraph event-marker button at get-up time. The performance of activity-based PM was scored as correct if the event-marker button was pressed within 15 min from morning wake-up or incorrect if the intention was executed more than 15 min after morning wake-up or not executed at all. For each participant, the percentage of correct execution of the PM task, based on the number of days of data recorded, was then computed. The group of children with ADHD obtained a percentage of accuracy (85%) very similar to the group of adults with ADHD (88%) and the group of adult healthy controls (85%) [18]. Such a measure of performance is quite rough; in other words, within the time interval of 15 min, some people may press the event-marker button early, while others may do it late. As a consequence, possible qualitative differences between groups could be masked.

For this reason, we chose to reanalyze previously collected data aiming to better explore the feature of activity-based PM performance in a naturalistic setting in a population of ADHD patients (children and adults). We aimed to check whether ADHD and age can moderate performance in an activity-based PM task. We chose to investigate PM through a naturalistic activity-based task, originally introduced by our research group [19,20] and later used by others [21]. More specifically, we decided to consider only the correctly executed tasks checking how long, after sleep ended, it took participants to remember to perform the task of pushing the event-marker button. Indeed, we aimed to verify whether the intention, that was not executed within the requested time, may still have a different status in memory such as to allow a delayed recovery.

In general, cognitive performances after waking up in the morning can be affected by the phenomenon of sleep inertia (SI). SI refers to a complex psychophysiological phenomenon observed after morning awakening that can be described as the gradual recovery of waking-like status after a night of sleep [22]. SI can be assessed in different ways: EEG, behavior, cognition, and motor activity. Recent work [23] has shown that motor sleep inertia in the morning lasts just over an hour. Keeping this suggestion in mind, we decided to consider event-marker buttons pushed within the first 60 min after the morning awakening. At the same time, we also considered motor activity in the first hour after sleep end. In this way, we could correlate the activity-based PM performance with motor sleep inertia.

Therefore, the aims of the work are:

1 To verify any difference between ADHD patients and healthy controls;
2 To investigate if and how activity-based PM performance changes with age;
3 To investigate a possible relationship between activity-based PM performance and motor sleep inertia.

This is an innovative application of actigraphy in basic and clinical research. To the best of our knowledge, there are no studies examining the activity-based PM performance, in an ecological setting through actigraphy, in children and adult ADHD patients, nor that correlate it with motor sleep inertia. However, we may expect a lower activity-based PM performance in clinical samples compared to healthy controls, a poorer PM performance in children compared to adults as well as an association between lower motor sleep inertia (in terms of higher motor activity at the morning awakening) and better PM performance (in terms of shorter time interval, in minutes, between the morning awakening and the pressure of the event marker).

## 2. Materials and Methods

### 2.1. Participants

Participants belonged to the following groups: (1) childhood attention deficit hyperactivity disorder (C-ADHD): 22 patients, 4 females and 18 males, mean age (SD) = 8.77 (1.77), range 5–12; (2) adult ADHD (A-ADHD): 35 patients, 14 females and 21 males, mean age (SD) = 37.29 (12.23), range 19–59; (3) children healthy controls (C-HC): 92 participants, 57 females and 35 males, mean age (SD) = 10.13 (0.42), range 9–11; (4) adult healthy controls (A-HC): 95 participants, 57 females and 38 males, mean age (SD) = 27.93 (14.35), range 31–50. C-ADHD and C-HC came from the study by Tonetti et al. [24], A-ADHD from [25] and A-HC from [26]. All participants wore the Actiwatch AW64 actigraph (Cambridge Neurotechnology Ltd., Cambridge, UK) on the non-dominant wrist, for seven consecutive days.

Adult participants gave written informed consent prior to inclusion in the original studies; if underage, written informed consent was provided by parents. Original studies were approved by the Ethics Committee in charge. As regards the children sample, the study was approved by the Bioethics Committee of the University of Bologna (Bologna, Italy; report of 11 August 2013). With reference to the comparison between adults with ADHD and adult healthy controls, the study was approved by the Ethics Committee of the Health Board of Alto Adige (ethical committee report number 51–2015 of 20 May 2015) and the Bioethics Committee of the University of Bologna (ethical committee report number 2.8 of 24 August 2014).

### 2.2. Actigraphy

All participants used the same model of actigraph, the Actiwatch AW-64 (Cambridge Neurotechnology Ltd., Cambridge, UK). This device is equipped with an accelerometer, which is sensitive to movement by means of a piezoelectric accelerometer presenting a sensitivity of ≥0.05 g. Filters were set to 3–11 Hz, with a sampling frequency equal to 32 Hz.

Version 5.32 of the Actiwatch Activity and Sleep Analysis software (Cambridge Neurotechnology Ltd., Fenstanton, UK) was used to extract the raw motor activity counts, minute-by-minute, considering the minutes close to wake-up time (i.e., sixty minutes after sleep end). To avoid the possible confounding effect of weekends, for each participant, only work/school days were considered in the analyses.

### 2.3. Activity-Based Prospective Memory Task

All participants were required to remember to push the event-marker button at get-up time. With reference to the activity-based PM performance, in the current study, each actigraphic recording was visually inspected nightly to verify whether participants remembered to press the event-marker button at wake-up, and we considered only the awakenings in which participants carried out the required task within the first 60 min after sleep end. In those cases, we took note of the time elapsed between sleep end and the pushing of the event-marker button.

### 2.4. Statistical Analyses

To compare the frequency distribution of the time elapsed between wake-up time and the pressing of the event-marker button (Response Time) between ADHD patients and healthy controls, separately for children and adults, we performed an independent-samples Mann–Whitney U test. Moreover, to simultaneously compare the four groups, we performed an independent-samples Kruskal–Wallis test; in the case of a significant effect, pairwise comparisons of group were carried out.

To better detect possible qualitative differences between the groups, we combined the participants who pressed the event-marker button within the first two minutes after wake-up (compliant with the task performances) versus all the other cases (late performances). Then, we analyzed the distribution of compliant and late performances in ADHD and healthy control groups by means of the chi-square test.

To analyze the possible relationship between motor sleep inertia and activity-based PM performance, we used Functional Linear Modeling (FLM) [27]. This statistical framework, specifically developed for the analysis of actigraphic data, detects if and when a pattern (separately for each group) statistically differs according to a continuous variable, i.e., time elapsing between wake-up and event-marker button pressure, over the time interval defined by wake-up and sixty minutes after get-up time. The raw motor activity pattern of sleep-wake transition is substituted with a function by applying the Fourier expansion model and is then examined through a non-parametric permutation F-test in order to establish a significant relationship between motor activity and the time of activity-based PM.

The significant level was set at *p* < 0.05 for all analyses performed.

## 3. Results

### 3.1. Comparison between ADHD and Control Groups

**Figure 1:**
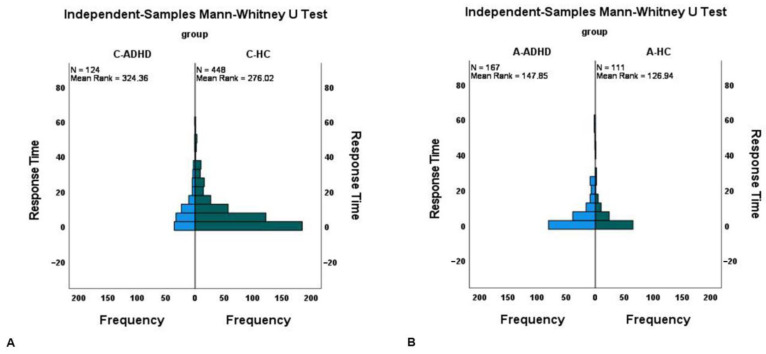
The frequency distribution of the Response Time calculated between the sleep end and the pressing of the event-marker button was presented in the Y axis of both graphs (**A**), on the left, for children sample and (**B**), on the right, for adult sample. The Response Time calculated as the difference between the sleep end and the pressing of the event-marker button (in minutes) represents the PM activity-based performance of the participants.

As regards the frequency distribution of the time interval between sleep end and the pressing of the event-marker button, it is possible to see that C-HC (mean rank = 276.02) are significantly denser (skewed to) near the wake-up time in comparison to C-ADHD (mean rank = 324.36) (U (N_C-HC_ = 448, N_C-ADHD_ = 124) = 32,470.5, z = 2.90, *p* = 0.004) (Figure 1A). The same trend is also observed comparing A-HC (mean rank = 126.94) and A-ADHD (mean rank = 147.85) (U (N_A-HC_ = 111, N_A-ADHD_ = 167) = 10663, z = 2.16, *p* = 0.03) (Figure 1B).

**Figure 2:**
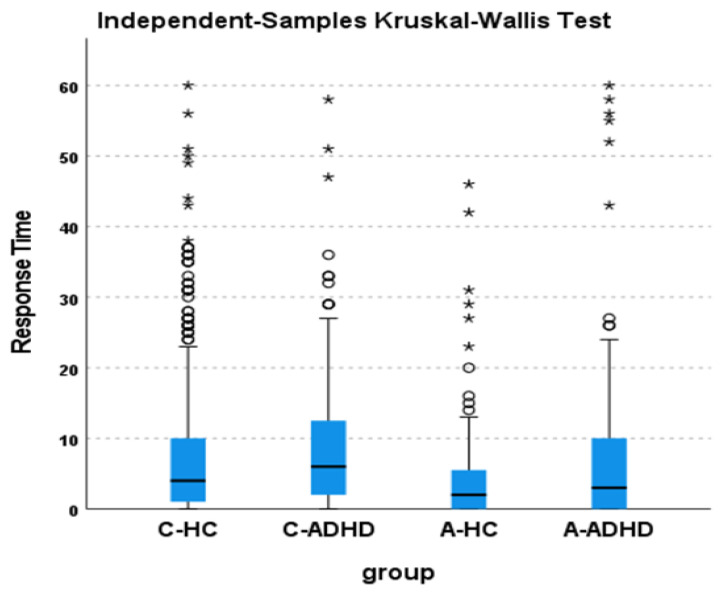
Boxplots of the Response Time between sleep end and pressing the event-marker button in C-HC, C-ADHD, A-HC, and A-ADHD groups. Box represents the interquartile ranges (IQR), that is, the range between the first quartile (Q1) and third quartile (Q3), the central line is the median (Q2). Whiskers indicate the dispersion of values below the first quartile and above the third quartile not classified as outliers (1.5 × IQR from the edge of the box). Circles represents the Mild outliers (Q3 + 1.5 × IQR), stars represent the extreme outliers (Q3 + 3 × IQR).

The comparison of all four groups was significant (χ^2^_3_ = 31.81; *p* < 0.001) (Figure 2). Performing pairwise comparisons of the groups (Table 1), it appears that the healthy control groups performed significantly better than the ADHD groups (except for the comparison between adult samples), and that adult groups executed significantly better in comparison to children’s groups (except for the comparison between A-ADHD and C-HC).

**Figure 3:**
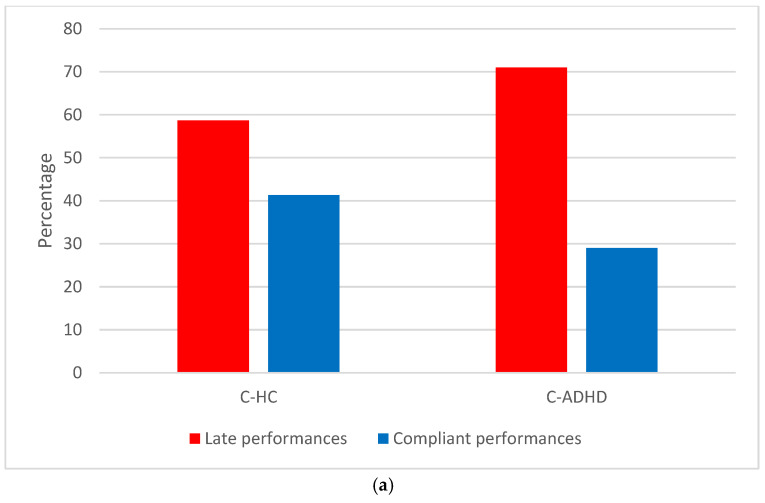

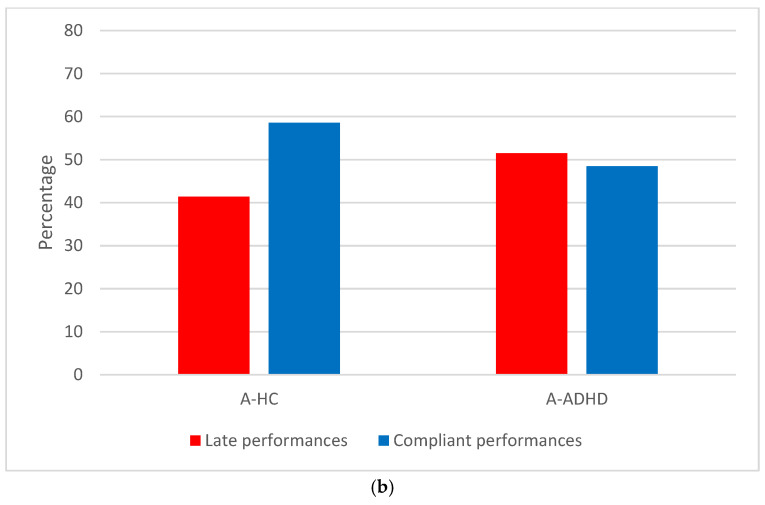
Distribution of children (**a**) and adults (**b**) in the HC and ADHD groups between compliant and late performances.

When we grouped participants according to the compliant performances (event-marker button pressed within 2 min of wake-up) and late performances (event-marker button pressed over 2 min after wake-up), we found a significantly different distribution between the C-ADHD (compliant performances 29%) and C-HC group (compliant performances 41.3%) (χ^2^_1_ = 6.16; *p* = 0.01) (Figure 3a), but no significant differences between the A-ADHD (compliant performances 48.5%) and A-HC group (compliant performances 58.6%) (Figure 3b) (χ^2^_1_ = 2.70; *p* = 1).

### 3.2. Motor Sleep Inertia and Prospective Memory Performance

**Figure 4:**
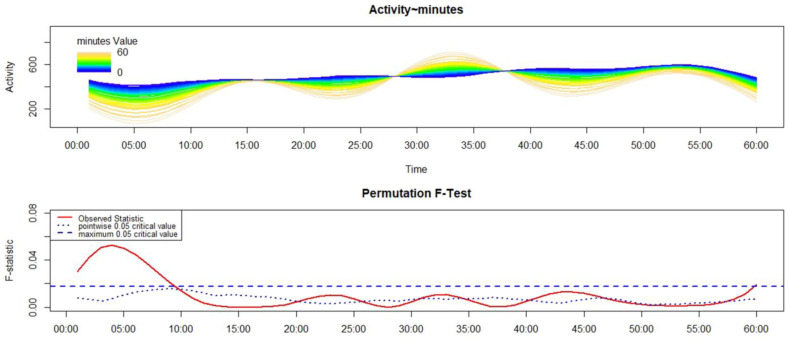
Results of FLM applied to the analysis of the variation of motor activity, within the first hour after wake-up, according to the time interval between sleep end and the pressing of the event-marker button in C-HC group. The upper panel shows the estimated activity pattern according to response time (in minutes), with different colors depicting the motor activity pattern over the first sixty minutes after awakening. Blue indicates the lowest response time, while yellow refers to the highest. Activity corresponds to the functional form of raw motor activity data. The lower panel shows the results of the non-parametric permutation F-test. Significant differences can be observed when the solid red line is above the dashed blue line.

As regards the relationship between motor sleep inertia and activity-based PM, we found a significant result only in the C-HC group. In particular, C-HC who pressed the event-marker button close to wake-up were significantly more active within 8 min of wake-up compared to those that remembered to perform the activity-based PM task later (Figure 4). For the C-ADHD group, there was no meaningful relationship.

In both of the adult samples, HC and ADHD, the relationship between motor sleep inertia and PM performance was not significant.

## 4. Discussion

This study had three main aims: (1) to examine potential differences in activity-based PM between ADHD and HC; (2) to investigate whether age can modulate the PM performance; (3) to explore the potential association between PM performance and motor sleep inertia. In general terms, we expected a poorer PM performance in ADHD than HC, a lower PM performance in children compared to adults, and lastly an association between higher degree of motor sleep inertia and lower PM performance.

As regards the first aim of the research, the results show that a significant performance difference in activity-based PM was present in the comparison between ADHD groups and control groups regardless of age, with ADHD patients performing worse than the control group. However, while further analyses confirmed the differences in the children’s groups, in the adult sample, the differences observed between ADHD and control groups no longer reached statistical significance. It is as if growth attenuated the performance gap in activity-based PM that characterizes individuals diagnosed with ADHD.

Our second aim was precisely to try to understand if and to what extent growth can modulate performance in activity-based PM tasks. As regards this aim, we observed an increase in activity-based PM performance in the adult groups compared to the children’s groups in both control and ADHD patients (Figure 2 and Table 1). It would therefore appear that the disadvantage shown by children with ADHD in comparison to healthy control children in activity-based PM tasks diminishes and eventually disappears in adulthood. Based on our data it is not possible to understand whether this improvement is linked to a development process of the nervous system or to the adoption of compensatory strategies or to both. Longitudinal studies, which are rarely conducted, would be desirable [28].

The third aim was to analyze a possible relationship between activity-based PM performance and motor sleep inertia. Basically, we expected that low motor sleep inertia, in terms of higher motor activity after morning awakening, was related to a better PM performance, in terms of a reduced time interval between T0 and T1. A significant result emerged only when analyzing PM performance of the C-HC group. In particular, participants who performed the activity-based prospective task in the first two minutes after awakening (excellent performances) showed high motor activation within the first ten minutes post awakening. It is possible to hypothesize that in healthy children, greater motor activation would result in a faster dissipation of sleep inertia and consequently a faster recovery of the prospective intention planned the night before. It is interesting that this result was not replicated in the C-ADHD group. A potential explanation of this pattern of results is that motor activity in the C-ADHD group is aspecific/dysfunctional and intrinsically related to the disorder. For this reason, we did not observe an association between motor activity and cognitive performance in this group; on the contrary, this association was found in the C-HC group, in line with previous literature summarized in the systematic review by Zeng and colleagues [29]. Overall, Zeng and colleague [29] underlined a relevant relationship between motor activity and cognitive development in children population. Moreover, it is possible to suggest that, with aging, such significant associations are weakened, potentially explaining the lack of a meaningful relationship in both adult samples. However, future research should study this aspect.

A general reflection that concerns all four experimental groups considered is that the percentages of excellent performances in general are rather low, despite the fact that the activity-based task is considered the easiest among the prospective tasks. This result could be interpreted by considering the condition in which the prospective task is required. The intention was planned before going to sleep, and the execution had to be carried out immediately upon awakening. Upon awakening, a return to typical cortical wakefulness activation is held back by the Sleep Inertia phenomenon, which involves reactivation of different cortical regions at different times [30,31]. The electroencephalographic parameters show a posterior–anterior activation trend where the frontal–prefrontal pathways are activated last [32]. In the same way, neuroimaging data have shown that patients with ADHD have a marked hypoactivation of the frontoparietal network, which is considered key for prospective memory [33]. This could explain the lower performance observed in the ADHD groups when compared with the respective control groups.

Finally, one might wonder if the processes activating the compliant and late performances are the same. In other words, how long can the intention of the activity-based prospective remain active? In the psychology of memory, there is a phenomenon known as the Zeigarnik effect [34], named for the gestalt researcher who first described it in 1927. This consists in the tendency to remember interrupted activities better than those that have been successfully performed. In experimental research, the execution of a prospective task, be it time, event, or activity, is considered correct only if performed within the established time or according to the cue event. If a person does not remember to carry out an action at time X or in association with a certain cue, is the intention deleted from memory? According to the Zeigarnik effect, unfinished intention remains in a “suspended activation”. The intention remains active, and will be deleted only after completion of the planned action; in other words, the uncompleted intention determines a heightened accessibility of the prospective intention in declarative memory.

Our study involved a task that had to be performed after spontaneous awakening, and the execution was monitored by actigraphic recording. However, although the requirement was very precise and underlined as being extremely important, the task was performed, in a rather high percentage of cases, after a considerable delay, so an activity task performed after two minutes and one performed later after wake-up time cannot be considered in the same way. The intention was fulfilled in both cases, but the underlying cognitive mechanism could be different. The task performed late demonstrates that the intentional component of the prospective memory probably continues to have a privileged status of activation [35].

This cognitive condition assumes an important adaptive value, suggesting the hypothesis of different mechanisms underlying the correct execution of the tasks planned, especially in PM where there is no external cue and self-initiated processing is required (time-based and activity-based PM). In the case of a compliant performance (within two minutes), we can say that the participant remembered to execute the planned intention and therefore the prospective task was successful. When the action (pressing the actigraph) was performed later after waking up, the activity cue association link was probably no longer present, but the suspended intention facilitates its recovery, even if it is delayed. Therefore, the performance could be interpreted as a Zeigarnik effect associated with the intrinsic motivation that facilitated a self-generated prospective pop-up.

Among the limitations of the current study, the assessment of just one type of PM, namely activity-based PM, must be mentioned. Furthermore, due to the retrospective nature of this work, the potential use by participants of external aids, in order to remember to push the event-marker button at the get-up time, was not checked. Moreover, for the same reason, the age and gender variables are not perfectly balanced between the samples of ADHD patients and healthy controls. Finally, the threshold for discriminating between compliant and late performance is partially arbitrary.

## Figures and Tables

**Table 1:** Statistics of the pairwise comparisons of group carried out to examine the significant group effect (four levels) in the time interval between sleep end and the pressing of the event-marker button.

Sample 1–Sample 2	Test Statistics	Std. Error	Standard Test Statistic	Sig. *a*
**A-HC–A-ADHD BOLD**	−64.80	29.86	−2.17	0.180
**A-HC–C-HC**	100.99	25.85	3.91	0.001
**A-HC–C-ADHD**	171.94	31.86	5.40	0.000
**A-ADHD–C-HC**	36.19	22.10	1.64	0.609
**A-ADHD–C-ADHD**	107.14	28.90	3.71	0.001
**C-HC–C-ADHD**	−70.95	24.74	−2.87	0.025

Significance values have been adjusted by Bonferroni correction for multiple tests.

## Data Availability

The data are not publicly available and cannot be shared due to ethical issues.
